# Editorial: Ecology of Amphibian-Microbial Symbioses

**DOI:** 10.3389/fmicb.2019.00766

**Published:** 2019-04-16

**Authors:** Eria Alaide Rebollar, Reid N. Harris

**Affiliations:** ^1^Center for Genomic Sciences, National Autonomous University of Mexico, Cuernavaca, Mexico; ^2^Department of Biology, James Madison University, Harrisonburg, VA, United States

**Keywords:** microbiota, amphibians, chytridiomycosis, symbiosis, antifungal bacteria

Symbiotic interactions among microorganisms and animals are ubiquitous in nature. Recent studies on amphibians have identified bacterial communities that establish symbiotic relationships with their hosts. Specifically, skin bacteria are known to play a relevant role in protecting the host against pathogens such as *Batrachochytrium dendrobatidis* (Bd) and the more recently discovered *B. salamandrivorans* (Bsal). Many of these bacterial symbionts have been isolated in culture and have been shown to produce antifungal metabolites with the capacity to inhibit Bd growth *in vitro* and *in vivo*. These findings have led to the development of probiotics as a promising strategy against chytridiomycosis, the disease caused by Bd and Bsal, which has caused dramatic declines and extinctions of amphibian species worldwide. In this context, the protective role of the skin microbiota, in addition to and likely in concert with the host immune responses, may be fundamental to the survival of amphibians that encounter emerging infectious diseases.

The field of amphibian microbial ecology started in earnest about 12 years ago, and it has greatly advanced since the development of next generation sequencing technologies and bioinformatic tools. The work published to date has shown that skin bacterial communities can be influenced by host species, host life-history stage, environmental conditions, surrounding bacterial communities that serve as reservoirs and external biotic agents such as pathogens. In addition to the bacterial component, the skin microbiome is also composed of symbiotic fungi that may also be protective, but whose role largely remains to be determined. Moreover, amphibians harbor symbiotic microbiota within their digestive tract, and there is growing evidence that these microorganisms may also play an important role for their hosts' health.

As the ecology of amphibian-microbial symbiosis is a relatively new field, there are still many unanswered questions such as: How stable are skin communities over time, and how resilient are they to perturbations? How do hosts influence the structure and function of these communities? What are the interactions occurring within these communities? And how do skin microbiomes function to protect amphibian hosts from pathogens, and under what conditions do pathogens change microbial community structure? Are key microbial species adapted to live on amphibian skins?

The aim of this Research Topic is to highlight recent research on amphibian microbiomes that addresses relevant questions on the ecology of amphibian-microbe interactions. The 21 publications gathered in this Research Topic have expanded our knowledge on the role of microbial symbionts of amphibians and have revealed novel insights that can direct the next set of research questions.

Many of the studies published in this Research Topic are based on field surveys. This is to be expected in a scientific discipline where the ability to characterize communities with culture-independent methods has only recently been possible. We are just now learning what amphibians' microbial communities look like and what may be affecting community structure. Studies that describe skin microbiota in natural settings aimed to evaluate how environmental factors, microhabitat conditions or host genetic traits influence the skin community diversity.

Specifically, Hernández-Gómez et al. evaluated how skin bacterial community structure of Eastern Hellbenders (*Cryptobranchus alleganiensis alleganiensis*) varied across sites. The authors found a high OTU turnover with changes in latitude and elevation, suggesting that environmental factors have a strong effect on skin microbiota. In addition, they found that skin community divergence was positively correlated with host population genetic divergence. Bird et al. evaluated the effect of environmental bacteria and host phylogenetic relatedness on two terrestrial salamander genera from the family Plethodontidae. The authors found that environmental factors were likely playing a more significant role in salamander cutaneous microbiome assemblages than host-specific traits. On a broader geographic scale, Bletz et al. determined that the microhabitat (arboreal, terrestrial, or riparian) is the strongest predictor of skin bacterial community structure on 89 frog species across 30 sites in Madagascar, with host phylogeny explaining less but significant portions of the observed variation.

Additional studies contribute to the descriptions of skin microbiota in tropical amphibians: Abarca et al. found that skin bacterial community structure of the Cane Toad (*Rhinella marina*) differed between Costa Rica and Puerto Rico. Moreover, Abarca et al. described the skin microbiota of 12 species of frogs at La Selva Biological Station in Costa Rica and found clear differences in skin microbial composition among different host species. Varela et al. analyzed the skin microbiota of three dendrobatid species in Panama and found that bacterial structure and predicted function may be influenced by environmental variables such as soil pH and precipitation. Additionally, Familiar-López et al. analyzed the skin bacterial communities of the cryptic frog *Philoria loveridgei* in tropical Australia. The authors found a strong effect of temporal variation (two years of sampling under distinct precipitation regimes) on the bacterial community structure and on the presence of Bd. They identified several OTUs whose relative abundances were significantly correlated with the presence of the pathogen. These studies confirm that species-specific and site-specific factors as well as environmental factors are involved in shaping skin microbial communities.

Many studies in this Research Topic focused on analyzing host-pathogen-bacteria interactions through different approaches including field studies, bacterial culturing and experiments. In a field survey of three species of frogs in tropical Australia, Bell et al. found that the upper limit of Bd infection intensity was negatively correlated with the number of inhibitory bacteria suggesting a protective role of the bacterial community. Catenazzi et al. found in a study of 28 frog species in the Peruvian Andes that the proportion of anti-Bd isolates was negatively associated with susceptibility to Bd, again suggesting a protective role of an anti-Bd bacterial community. In an experimental study, Robak and Richards-Zawacki found that bioaugmentation with an inhibitory bacterial isolate led to longer survival of a Bd infection at 14°C, but not at 26°C, demonstrating the important role of temperature in the bacterial community's protective function. However, in an experimental study, Jani and Briggs found that the effect of Bd on the skin microbiome of *Rana sierrae* from the western US was greater than the effect of microbial community structure on Bd infection intensity. Thus, the skin microbiota can have a protective function, although if the pathogen Bd is able to achieve wide-spread infection, then Bd can alter bacterial community structure. In addition, the authors determined a clear effect of the host and the aquatic environment on skin microbial structure.

Anthropogenic factors in the environment may have a negative effect on the protective nature of the microbiota. Huang et al. found evidence that the gut microbiota of frogs in degraded habitats differed from the microbiota in a natural habitat, and these differences could be related to disease susceptibility. The effect of pesticides discussed in McCoy and Peralta will likely cause alterations on host-bacteria-pathogen interactions. Thus, this topic requires further investigations that will be particularly relevant to amphibian species that are exposed to human activities such as agriculture and cattle raising.

Several studies evaluated pathogen-bacteria or bacteria-bacteria interactions in order to inform probiotic development. Probiotics rely on our capacity to isolate in culture a high proportion of bacteria. In this respect, Medina et al. evaluated the proportion of culturable bacteria using different media and determined that a high proportion of the skin community on American toads (*Anaxyrus americanus*) is culturable and that these bacteria include antifungal bacteria independent of isolation media. In an experiment involving the gut microbiota of an Asian tropical treefrog (*Polypedates megacephalus*), Weng et al. obtained interaction networks from 16S amplicon sequencing data, and the interactions were validated through bacterial inoculations. The authors showed that as opposed to single strains, a combination of cooperative microbes yielded a higher relative abundance of probiotic bacteria and did not have negative effects on the acquired immune system. Muletz-Wolz et al. found that *Batrachochytrium* strains (including Bsal) and temperature interact to determine the ability of bacteria to inhibit the pathogen, again highlighting the importance of temperature as shown by Robak and Richards-Zawacki. Bacteria that inhibit the pathogens over a range of temperatures would be preferred probiotics. Bletz et al. also found that bacterial inhibition could be a function of Bd genotypes, but that some bacterial species showed broader spectrum inhibition against all tested Bd genotypes and that these OTUs would be better probiotic candidates. Kueneman et al. characterized skin-associated bacterial and micro-eukaryotic diversity and used a network analysis to identify species that inhibited Bd and thus could be probiotic candidates. Kearns et al. presented evidence that anti-Bd fungal species could be effective probiotics while not negatively affecting responses of the acquired and innate immune systems as some bacterial species can do. These last two studies emphasize the need to study skin fungi as important microorganisms present in amphibian skin microbiomes.

Omics methods (metagenomics and transcriptomics) are starting to be used to study microbial function and will certainly expand our knowledge on host-microbe interactions. Rebollar et al. used a shotgun metagenomic approach and found that the skin of *Craugastor fitzingeri* contained bacteria with a wide variety of genes that code for secondary metabolites, which may be involved in bacterial-bacteria communication and bacteria-host interactions and which could in addition protect the host against fungal pathogens. Madison et al. used transcriptome sequencing of the antifungal skin resident, *Serratia marcescens*, and demonstrated that key genes were up- and down-regulated in response to Bd presence *in vitro*.

In summary, this Research Topic has compiled a variety of studies that address relevant questions about symbiotic microbiomes in amphibians with particular emphasis on their role in pathogen protection. These studies used distinct approaches to analyze skin and gut microbial diversity such as culturing, 16S and I8S amplicon sequencing, shotgun metagenomics and transcriptomics. Overall, it is becoming clear that different abiotic and biotic factors shape skin microbial communities and that host-bacteria-pathogen interactions are dependent upon many of these factors. We suggest that soon the field will move from the basic (and necessary) descriptions of microbial communities to more experimental approaches that include the use of omics methods and a variety of novel analytic and multivariate approaches. In addition to providing more insights into the microbial and disease ecology of amphibians, these studies may lead to effective ways to manipulate the microbiome to achieve protection from diseases.

## Author Contributions

ER and RH contributed equally to the proposal and editorial work of this Research Topic. Both ER and RH equally contributed to the writing of the Editorial.

### Conflict of Interest Statement

The authors declare that the research was conducted in the absence of any commercial or financial relationships that could be construed as a potential conflict of interest.

